# The *Pearl River Declaration*: a timely call for enhancing health security through fostering a regional one health collaboration in the Asia-Pacific

**DOI:** 10.1186/s12992-020-00606-y

**Published:** 2020-09-07

**Authors:** Noore Alam, Cordia Chu, Qianlin Li, Allison Crook, Maxine Whittaker, Tjandra Aditama, Elena Schak, Dicky Budiman, Bonnie Barber, Jiahai Lu

**Affiliations:** 1 Centre for Environment and Population Health, Griffith University, Nathan, Australia; 2 Queensland Health, Queensland Government, Brisbane, Australia; 3 One Health Research Centre, School of Public Health, Sun Yat-sen University, Guangzhou, Guangdong Province China; 4 Department of Agriculture and Fisheries, Queensland Government, Brisbane, Australia; 5 James Cook University, Townsville, Australia; 6 World Health Organization, New Delhi, South-East Asia Region India

**Keywords:** Health security, Emerging infectious disease, Antimicrobial resistance, One health, Transdisciplinary collaboration, China

## Abstract

The Second International Symposium on One Health Research (ISOHR) was held in Guangzhou city, China on 23–24 November 2019. A transdisciplinary collaborative approach, One Health (OH), was the central theme of the symposium which brought together more than 260 experts, scholars and emerging researchers from human health, veterinary health, food safety, environmental health and related disciplines and sectors. More than 50 organizations including World Health Organization, Centers for Disease Control (USA), and Queensland Government (Australia) participated in the symposium. Scholars, experts and emerging researchers, policy-makers and practitioners in their respective fields delivered over 50 presentations at the symposium, highlighting the collective vulnerability to some of the emerging health challenges the region was combating. These included emerging infectious diseases, antimicrobial resistance, climate change, food safety and the growing burden of non-communicable diseases. The *Pearl River Declaration*, emanated from the symposium, called for establishing a One Health Cooperation Network in the Southeast Asia–Pacific region with a vision to strengthen regional health security through sharing each other’s knowledge and experience, and making investments in workforce development, scientific innovations such as vaccine research and development, sharing epidemic intelligence, risk identification, risk communication and appropriate response measures against emerging health threats.

## Introduction

Emerging infectious diseases, antimicrobial resistance, climate change, food safety and security, and the growing burden of non-communicable diseases were identified as some of the most pressing challenges to global health security at the Second International Symposium on One Health Research (ISOHR) held in Guangzhou, China on 23–24 November 2019. The symposium was jointly organized by Sun Yat-sen University (China), Griffith University (Australia), South China Agricultural University (China) and Guangzhou Medical University (China). A transdisciplinary collaborative approach, known as One Health, was identified as a key opportunity to promote human, animal and environmental health within and between countries around the world [1]. The symposium sought to facilitate a shared understanding of transdisciplinary synergies and the need for a multi-sectoral collaboration in research and practice to tackle the growing threats to global health security.

The ISOHR brought together more than 260 experts, scholars and emerging researchers from China and around the world in as diverse areas as public health, clinical medicine, veterinary medicine, laboratory science, food safety, environmental health, agricultural science, military medicine, health systems and policies. More than 50 organizations including multiple Chinese Government departments and research institutions, and representatives from World Health Organization, Centers for Disease Control (USA), and Queensland Government (Australia) participated in the symposium.

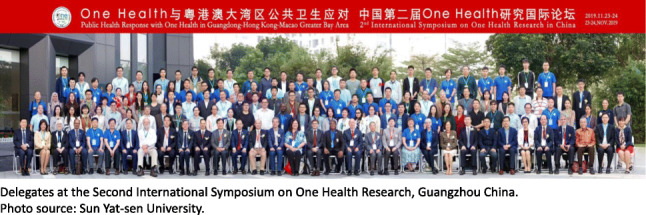



Over the past two centuries the world has witnessed spectacular innovations and successes in public health and medical science. The most notable among those were the invention of vaccines (1796 AD) and antibiotics (1928), the eradication of small pox (1979) and the introduction of a simple, low-cost oral rehydration therapy (sugar-salt-clean water solution) for treatment of dehydration caused by diarrhea. Collectively, these innovations have saved tens of millions of lives [2–4]. These achievements may have provided a sense of complacency to some for wining over epidemic diseases. Ironically, this complacency was challenged by the emergence of some of the deadly diseases such as human immunodeficiency virus (HIV) in the 1980s, severe acute respiratory syndrome (SARS) in 2002 [5], and lately, the 2019 coronavirus disease (COVID-19).

The novelty of the virus, the nature and speed of the interspecies and cross-border transmission of COVID-19 prompted World Health Organization to declare it a ‘Public health emergency of international concern’ [6], whereas SARS prompted the overhaul of the global health governance mechanisms resulting in the *International Health Regulations 2005* [7].

## Infectious diseases

The ISOHR observed with a great concern that over the past five decades, the world has witnessed the emergence of about 40 new diseases including Legionnaires’ disease, HIV/AIDS, hepatitis C, mad-cow disease, SARS, Middle East Respiratory Syndrome (MERS), Nipah and Ebola virus diseases. Twenty of these diseases were found in China alone [8]. Many of these new diseases along with the existing climate-sensitive vector-borne diseases such as dengue, Zika and West Nile virus, remain ongoing threats to health security worldwide [9]. Many emerging diseases, after a period of absence or being controlled, re-emerge as epidemic with devastating impacts. For example, dengue in Bangladesh in 2019 [10], measles in the Pacific since 2017 [11] and polio in the Philippines in 2019 [12]. Malaria, multidrug-resistant tuberculosis, HIV/AIDS, neglected tropical diseases and viral hepatitis affect billions of people worldwide, and are responsible for more than 4 million deaths annually, where the vast majority are in low- and middle-income countries (LMICs) [13]. The ISOHR noted that approximately 75% of emerging diseases originate in animals (zoonoses) with greater frequency of outbreaks and significantly higher burden in LMICs than high-income countries [14–17].

The ISOHR acknowledged the role of economic globalization in the twenty-first century and the associated growth in tourism and trade. These and other developments have significantly increased the mobility of populations and trade in goods worldwide and increased the risk of infectious disease spread across regions and countries. In some areas this has resulted in a continuing epidemic and pandemic threat. The impact of global climate change, agricultural intensification, rapid economic growth combined with population growth and urbanization, further complicate public health challenges globally and more specifically in LMICs [18]. Today, more than half of the world’s 7.7 billion population live in the South Asia and South-East Asia—a region which includes China, India, Indonesia, Bangladesh, Pakistan and Vietnam [19]. This densely populated region hosts some of the fastest growing economies in the world, and faces a wide range of challenges including climate change, food safety and emerging infectious disease threats. Many of these countries are often dubbed as ‘emerging disease hotspots’. [20]

In the recent past, the region has experienced some of the deadliest epidemics resulting in formidable losses not only to health but also impacting heavily on their economies with substantial sociological and political repercussions [20]. For example, after the 2002–03 SARS pandemic [21], which caused enormous losses to health and economies of over 30 countries, the highly pathogenic avian influenza (H5N1) has become endemic in parts of China and other countries in the region including Bangladesh, Indonesia, and Vietnam, and in parts of Africa [22]. The combination of avian and human strains in a favorable environment can result in a sustained spread from birds to human, and then human to human with a potential for a pandemic causing large-scale illnesses and deaths worldwide. There is an overwhelming body of evidence that infectious disease occurs at human-animal-environment interface and that their transmissions and the magnitude of impacts are influenced by many factors including behavioral, social, environmental, and cultural [20]. These recognitions have been the catalyst to call for a broad-based, holistic approach, known as One Health (OH), which is defined as, “The collaborative effort of multiple health science professions, together with their related disciplines and institutions – working locally, nationally, and globally – to attain optimal health for people, domestic animals, wildlife, plants, and our environment” [23].

Countries increasingly recognize the value of working collaboratively and sharing knowledge, expertise and resources for the protection of their own country and to address the collective vulnerability in today’s globalized world. This recognition is evidenced by the Australian Government’s regional health security initiative known as ‘Indo-Pacific Centre for Health Security’ established in 2017 [24]. The initiative is focused on a holistic, transdisciplinary and multisectoral approach, known as “One Health” (OH), to strengthen the health security capacity across the Indo-Pacific region [24].

## Antimicrobial resistance

The ISOHR noted with concern that the growing antimicrobial resistance (AMR) was a significant threat to the core of modern medicine as it seriously hampers the efficacy and sustainability of an effective public health response to infectious diseases. The complex root of the problem and the process of widespread, systematic misuse of antibiotics in human and animal medicine, and in food production, demands systematic and coordinated action involving multiple sectors, and disciplines beyond health [25]. The unregulated and often uncontrolled use of antibiotics in human medicine as well as the overuse of non-therapeutic antibiotics in animals for food production compromises the health of the public and challenges health systems globally. The intersection between veterinary sanitary, food safety and public health is a highly regulated area, particularly across the European Union which has a legislative basis for action against AMR [26]. A regional collaboration such as the proposed One Health Cooperation Network in the Southeast Asia and the Pacific region, as pronounced at the ISOHR’s call, could play a pivotal role in creating a similar legislative environment in the region as well as enhancing a holistic, transdisciplinary response to AMR.

## Chronic diseases

Infectious diseases and AMR are not the only causes of concern for the global health security. Similarly, the scope of OH is not limited to infectious disease only. The growing burden of chronic disease, the double burden of malnutrition which is defined as the coexistence of overnutrition (overweight and obesity) and undernutrition (stunting and wasting) [27], climate change and its impacts are some of the emerging threats all countries are facing in one way or another.

## The one health approach

Infectious disease transmission occurs at the human-animal-environment interface. The magnitude of the impact of disease and its transmission is influenced by many factors including human behavior, and society, the environment, and the culture of the community. Thus, a broad-based, holistic approach, known as One Health (OH), has received prominence in recent years. Countries increasingly recognize the collective vulnerability to emerging health security threats such as epidemics and pandemics and seek to forge joint effort for mutual benefit. One example of such collaboration effort is the Australian Government’s regional health security initiative, the ‘Indo-Pacific Centre for Health Security’. [24] Established in 2017, the initiative focuses on a holistic, transdisciplinary approach, “One Health” (OH), to strengthen the health security capacity across the Indo-Pacific region. The ISOHR called for a OH approach to collaborative research and practice which would provide opportunities for better understanding of emerging health risks, and avenues for collaborative and coordinated action for prevention through risk identification, risk communication, sharing epidemic intelligence and appropriate control measures. While the focus of OH has been traditionally on infectious zoonotic diseases, its scope and application can be as diverse as agricultural production and land use to climate change, biodiversity and environmental health to food safety––anything that transects the human, animal and environments, thus opportunities for maximizing the benefits of OH approach [28].

## Strengthening the one health approach in the Asia-Pacific region

Looking through the lens of etiology, infectious diseases are not only caused by bacteria, viruses and parasites. They are also influenced or shaped by social, economic, political, legal and cultural conditions. Thus, the breadth of emerging health issues and the application of OH to address those will go much farther than the traditional health outlook.

In line with its fast-paced economic development, China remains focused on combating emerging health threats including emerging infectious diseases such as SARS and the coronavirus disease (COVID-19), and controlling the spread of antimicrobial resistance [8, 29]. Having learnt the lessons from SARS, including the application of a holistic OH approach, China was able to successfully counter some of the potentially pandemic diseases such as H5N1, H1N1 and H7N9 [8]. By making sustained, incremental investments in health, China is pioneering through its efforts in promoting the OH approach to research and practice to address emerging health challenges of the twenty-first century [28, 30]. In today’s globalized and increasingly complex world, emerging public health issues that transect international borders often become complicated issues and require international law [31]. The need for effective health diplomacy [32], both state-and non-state-based arrangements for multidisciplinary collaborative research, exchange of knowledge and information between sectors and disciplines within and among countries has thus never been greater [33].

In line with this acknowledgement, the Pearl River Declaration [28] (Table 1) called for establishing a One Health Collaboration Network encompassing countries across the greater South–Southeast Asia and the Pacific region with a vision to contribute to building a shared future for all of the world. Although the concept of OH and the value of its essence, collaboration, is generally accepted by relevant inter-related sectors and disciplines, its application in dealing with emerging health issues has often been described as limited, particularly in resource-limited settings [34]. To overcome those limitations, focused actions in four areas; legislation, communication, education and investment were recommended at the first International Symposium of OH Research in Guangzhou, China in 2014 [30]. Half a decade later, the need for continuous actions on those areas were resounded at the second ISOHR in 2019 [28].

**Table 1:** The Pearl River Declaration, Guangzhou, China, 24 November 2019

Five years ago, we gathered together in Guangzhou to discuss the way forward in bringing about One Health in China. Over the next 5 years, we held the first China One Health Symposium where we signed the *Yuexiu Mountain Declaration*, setting up the first One Health Research Center, and assembling the “China One Health League”, marking a substantial step forward for the One Health research in China. Since then, we have made great progress, with substantial efforts to accelerate the construction of an interdisciplinary, multisectoral, cross regional international collaborative platform for One Health, helping to move One Health from concept to practice. Although we have made great strides, much remains to be done.	
In this second decade of the twenty-first century, the world is undergoing complex changes that pose enormous challenges to human health worldwide. This is marked by the cross-species transmission of pathogens, intensive agriculture, the overuse of antibiotics, and environmental and food pollution. It has been reported that over 70 % of emerging infectious diseases are zoonoses, while multi-drug and super-drug resistant bacterial pathogens constantly emerge, and there are increasingly frequent food-borne diseases and food safety incidents. As China seeks to contribute to building a shared future for all of the world through its *Belt and Road initiative*, by supporting the construction of Greater Bay Area, and by enhancing economic globalization, it faces complex challenges from public health, veterinary health and environmental health, as well as food safety. These problems cannot be solved by single department or discipline. Thus. we must tackle the world’s health issues through the One Health approach.	
China’s One Health action has entered a new stage. We must now foster good regional leadership, and focus on expanding, developing and strengthening the One Health Cooperation Network in Southeast Asia and the Pacific. We can continue to improve this platform and make it more efficient by strengthening our ties within this region. This will enhance our ability to carry out comprehensive, cooperative research. China and Southeast Asia should coordinate their efforts to build a shared future, by establishing ongoing One Health training courses, jointly cultivating young talent, creating efficient information exchange networks, and sharing information and resources, so that together we can face and meet the world’s health challenges.	
Mountains and seas are no obstacle to people with the same aspirations. The development of China’s One Health benefits from full openness and active communication. In the future, communication and cooperation should be carried out at a higher level, with a broader scope and in a wider range of fields. We will firmly and steadily promote One Health to achieve concrete outcomes. With water-like persistence and kindness, we will encourage people’s trust in and support for One Health. We will learn from the international One Health movement and take its momentum forth, sharing its ideas to benefit our neighbors, and the world.	
We have only one planet, and we live together in one world. Let us work together to create ONE WORLD, ONE HEALTH.	

The process of initiating such a regional collaboration by Griffith University, a co-convener of the ISOHR, began as early as 2016 with the establishment of the Global Research Consortium on Risk Communication, Emergency Management and Adaptation to Climate Change for Health (REACCH). This was undertaken in partnership with 15 institutions including government departments across the region. REACCH members have contributed to joint research, policy advocacy and capacity-building to improve health systems and address collective vulnerability to emerging health threats.

The establishment of a cross-disciplinary Research and Policy Advocacy Hub for Global Health Security at Griffith University, Australia in 2019 further supports this overall regional objective. The Hub hosts two broad networks:

(i.) The One Health Research Network, established in 2016––a collaboration with One Health networks in China and Indonesia, focusing on diseases that occurs at human-animal-environment interface.
(ii.) The country programs network for Bangladesh, China, Indonesia, Mongolia, the Philippines and Vietnam, consisting of many institutional members from these countries guided by a Memorandum of Understanding of each with Griffith University, having been gradually expanded since 2006. These collaborations focus on health system policies and operational capacity building in the region, as well as emerging health challenges including climate change adaptation, disaster risk reduction, zoonotic and vector-borne disease control.

Among many tangible outcomes achieved through the establishment and function of these networks has been the delivery of over 10 public health leadership programs that have successfully nurtured more than 120 Fellows across the region. Many of these Fellows have gone on to leadership of the COVID-19 pandemic response in their respective countries over recent months [35].

## Conclusion

Addressing emerging health challenges is the collective responsibility of nations as they are collectively vulnerable in the globalized world. If health is for all, as it is often heard being said, then all should be working for health. The resounding message of the Pearl River Declaration was the need for greater commitment and collaboration among nations across the mountains and seas under the umbrella of OH for a future healthy global community. As important regional development partners in health in the Indo-Pacific region, countries such as Australia, China, Thailand, Singapore and international bodies such as Asian Development Bank and WHO can play a pivotal role in country-capacity building through sharing knowledge and experience, as well as making investments in workforce development, scientific innovations such as vaccine research and development. These can be better achieved through establishing a regional One Health Cooperation Network.

## Data Availability

No primary data collection was involved in this article. All secondary data presented in this article have been appropriately referenced to their original sources.
